# Effects of pressure-support ventilation with different levels of positive end-expiratory in a mild model of acute respiratory distress syndrome

**DOI:** 10.1186/2197-425X-3-S1-A573

**Published:** 2015-10-01

**Authors:** PAF Magalhães, GDA Padilha, L Moraes, CL Santos, LB de Andrade, MGPdA Magalhães, MDCMB Duarte, PRM Rocco, PL Silva

**Affiliations:** Federal University of Rio de Janeiro, Rio de Janeiro, Brazil; Professor Fernando Figueira Mother and Child Institute, Recife, Brazil

## Introduction

Pressure-support ventilation improves lung mechanics, blood gas exchange, hemodynamics, and work of breathing (WOB) in mild acute respiratory distress syndrome (ARDS) [1, 2]. Nevertheless, those beneficial effects could be dependent of positive end-expiratory pressure (PEEP) applied during mechanical ventilation. So far, no study has compared pressure-support ventilation (PSV) with pressure controlled ventilation (PCV) in different PEEP levels.

## Objective

To compare PSV and PCV target to protective tidal volume (V_T_=6ml/kg) using two PEEP levels (2 and 5 cmH_2_O) in a mild ARDS model.

## Methods

Thirty-two male Wistar rats (310 ± 19 g) were submitted to intratracheal *Escherichia coli* lipopolysaccharide (200µg in 200µl of saline) instillation. After 24 hours, animals were anesthetized, tracheotomized, and their lungs were mechanically ventilated in PSV to achieve V_T_ = 6 ml/kg. After baseline data collection, animals were randomly divided to four groups (n=8/group):

1. PCV + PEEP = 2 cmH_2_O (PCV-P2);
2. PCV + PEEP = 5 cmH_2_O (PCV-P5);
3. PSV + PEEP = 2 cmH_2_O (PSV-P2);
4. PSV + PEEP = 5 cmH_2_O (PSV-P5).

Animals were ventilated for 2 hours. Mean arterial pressure (MAP), arterial blood gases, peak airway (Ppeak,_RS_) and peak transpulmonary (Ppeak,_L_) pressures, and pressure-time product (PTP), as a surrogate of WOB, were evaluated.

## Results

All animals showed better oxygenation along time, regardless of ventilator strategy. Animals submitted to PCV, regardless of PEEP, received more colloids to keep MAP>70 mmHg. Ppeak,_RS_, and Ppeak,_L_ were higher in animals submitted to PEEP = 5 cmH_2_O than PEEP = 2 cmH_2_O, independently of pressure-controlled, and pressure-support ventilator strategies. Nevertheless, at PEEP = 5 cmH_2_O, but not at PEEP = 2 cmH_2_O, animals submitted to PSV showed lower Ppeak,_RS_, and Ppeak,_L_ compared to PCV animals (PSV-P5:11.2 ± 1.9 cmH_2_O *vs* PCV-P5:15.3 ± 1.4 cmH_2_O, p < 0.05). In accordance, PTP was lower in animals submitted to PEEP = 5 cmH_2_O compared to PEEP = 2 cmH_2_O during PSV (PSV-P5:0.08 ± 0.03 cmH_2_O.s *vs* PSV-P2:0.22 ± 0.09 cmH_2_O.s, p < 0.05).

## Conclusion

In a mild ARDS model, pressure-support ventilation is associated to better hemodynamics, lung mechanics, and it seems to have a dependent effect of the adjusted PEEP level, as depicted by work of breathing.

## Grant Acknowledgment

CNPq, FAPERJ, CAPES, PRONEX, MS-DECIT
